# Using Implementation Theories to Tailor International Clinical Guidelines for Post-Stroke Gait Disorders

**DOI:** 10.3390/healthcare13151794

**Published:** 2025-07-24

**Authors:** Salem F. Alatawi

**Affiliations:** Department of Health Rehabilitation Sciences, Faculty of Applied Medical Sciences, University of Tabuk, Tabuk 71491, Saudi Arabia; sfalatawi@ut.edu.sa

**Keywords:** national clinical guidelines, knowledge to action, gait, stroke, tailoring, implementation

## Abstract

**Background/objective**: Tailoring involves adapting research findings and evidence to suit specific contexts and audiences. This study examines how international stroke guidelines can be tailored to address gait issues after a stroke. **Methods**: A three-phase consensus method approach was used. A 10-member health experts panel extracted recommendations from three national clinical guidelines in the first phase. In the second phase, 362 physiotherapists completed an online questionnaire to assess the feasibility of adopting the extracted recommendations. In the third phase, a 15-physical therapist consensus workshop was convened to clarify factors that might affect the tailoring process of the extracted recommendations of gait disorder rehabilitation. **Results**: In phase one, 21 recommendations reached consensus. In the second phase, 362 stroke physiotherapists rated the applicability of these recommendations: 14 rated high, 7 rated low, and none were rejected. The third phase, a nominal group meeting (NGM), explored four themes related to tailoring. The first theme, “organizational factors”, includes elements such as clinical setting, culture, and regulations. The second theme, “individual clinician factors”, assesses aspects like clinical experience, expertise, abilities, knowledge, and attitudes toward tailoring. The third theme, “patient factors”, addresses issues related to multimorbidity, comorbidities, patient engagement, and shared decision-making. The final theme, “other factors”, examines the impact of research design on tailoring. **Conclusions**: Tailoring international clinical guidelines involves multiple factors. This situation brings home the importance of a systematic strategy for tailoring that incorporates various assessment criteria to enhance the use of clinical evidence. Future research should investigate additional implementation theories to enhance the translation of evidence into practice.

## 1. Introduction

Clinical practice guidelines (CPGs) are essential resources for healthcare professionals and patients aiming to make informed treatment decisions grounded in empirical research [1]. The development of these guidelines follows a structured process that includes several crucial steps: identifying the clinical problem, assembling a multidisciplinary team for guideline development, forming a systematic review team, and conducting a literature review to identify the most reliable research evidence and pertinent practice experiences [2].

Many countries have developed National Clinical Guidelines (NCGs) for stroke management. These guidelines are important for guaranteeing standardized care and enhancing outcomes for individuals affected by stroke [3]. Examples of countries that have established their guidelines include the United States (the American Heart Association (AHA) and the American Stroke Association (ASA)) [4,5], the United Kingdom (the National Institute for Health and Care Excellence (NICE)) [6], and Canada (the Canadian Stroke Best Practices Recommendations) [7]. However, these CPGs encompass a wide range of stroke-related issues, which may hinder the effective implementation of these guidelines in clinical practice [1,2].

Stroke is the leading cause of long-term disability and the second-leading cause of death worldwide [1]. Gait disorders often arise after a stroke and can significantly affect an individual’s mobility, independence, and overall quality of life. It is estimated that nearly 60–80% of stroke survivors experience gait disorders [2].

Stroke is the leading cause of long-term disability and the second-leading cause of death worldwide [8]. Gait disorders are among the most common clinical conditions that arise after a stroke [8]. Nearly 60–80% of individuals who have suffered a stroke experience impairment in their gait [8]. However, despite national guidelines providing evidence for healthcare interventions addressing gait disorders post-stroke, their practical implementation often falls short [1]. Consequently, a gap exists between the development of evidence-based best practices and their implementation in everyday clinical settings.

The vascular topography of acute stroke refers to how blood vessels are affected and which parts of the brain suffer from reduced blood flow or bleeding. Understanding this topography is essential for evaluating the stroke’s impact on motor functions, especially regarding gait disorders [9]. Gait disorders, such as hemiparesis, ataxia, and bradykinesia, are frequently observed in individuals after a stroke. However, there is an intricate connection between the vascular topography of acute stroke and these gait disorders [9].

Understanding the affected areas of the brain can guide treatment strategies and enhance recovery outcomes for individuals facing gait issues related to stroke. A clinical report identified lacunar stroke as the primary subtype of infarction occurring in the territory of the posterior cerebral artery (PCA) [9]. The study also found that certain symptoms were more common in stroke patients with PCA infarction than in those with ischemic strokes from the middle cerebral artery (MCA) and anterior cerebral artery (ACA) [9].

The NCGs provide substantial evidence regarding gait disorders following a stroke. However, implementing gait CPGs can be challenging for several reasons [1,2]. The authors [10,11,12] suggest that many guidelines are not adequately tailored to meet the specific needs of individual patients. They point out that variability in factors such as age, mobility levels, and specific injuries necessitates personalized approaches, which standardized guidelines often lack.

Implementing international clinical guidelines can be challenging due to various factors. The literature identifies several common challenges to the successful implementation of these guidelines, including variability in healthcare systems, cultural differences, resource limitations, resistance to change, lack of awareness or knowledge, training and education needs, inconsistent interpretation, patient engagement, and issues related to policy and funding [12,13]. The authors concluded that overcoming these challenges requires a multi-faceted approach that includes education, resource allocation, and the promotion of a culture of evidence-based practice. Additionally, collaboration among stakeholders—such as healthcare providers, policymakers, and patients—is crucial for effective implementation [12,13].

These challenges may redirect focus to national clinical recommendations, which offer a detailed examination of the evidence supporting healthcare interventions [11,12,13]. Researchers have noted a possible delay in adherence to these recommendations, despite the growing amount of data available [8,9,10]. Consequently, there exists a gap between the evidence currently available and its application in daily practice [12,13,14].

Implementation research explores strategies that connect evidence with practice, thereby improving the relevance and applicability of this evidence [14]. Current literature indicates that our understanding of the methods for implementing evidence-based practices might be limited [14]. Evidence implementation frameworks are increasingly available, and this field is progressing rapidly. Notable examples of these frameworks include the Ottawa Model of Research Use [15], PARIHS [16], and KTA [17].

**Figure 1:**
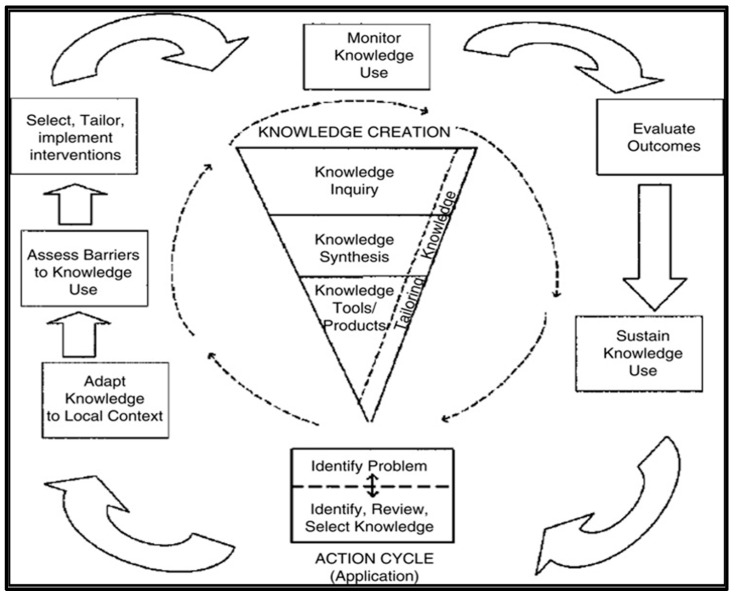
Knowledge of action framework (Graham et al., 2006) [17]. It includes two main components: (1) Knowledge creation (represented by the funnel) involves knowledge inquiry, knowledge synthesis, and knowledge tools/products. (2) The action cycle includes seven components designed to facilitate the translation of knowledge into practice.

The Knowledge-to-Action (KTA) framework encompasses both knowledge creation and action (Figure 1). This theoretical framework utilizes the knowledge funnel/triangle as a metaphor for the process of knowledge filtration, also referred to as tailoring. Throughout the knowledge creation process, tailoring results in synthesized information that may be more advantageous to end consumers. According to Graham et al. (2006) [17], tailoring facilitates collaboration between researchers and knowledge users, allowing them to share insights and develop solutions together [17]. Furthermore, this approach has the potential to impact professional practice.

The KTA framework effectively demonstrates how national clinical guidelines can function as a funnel or triangle for knowledge creation. However, tailoring—an essential element of knowledge creation within the KTA framework—has proven to be a significant challenge. Consequently, the primary aim of this study was to employ a technique that enhances understanding of the tailoring process and its practical application in stroke rehabilitation. The research question looked at how “tailoring” can be used in the funnel structure of the KTA framework to create recommendations based on different international clinical guidelines for gait disorders after a stroke.

## 2. Methods

**Figure 2:**
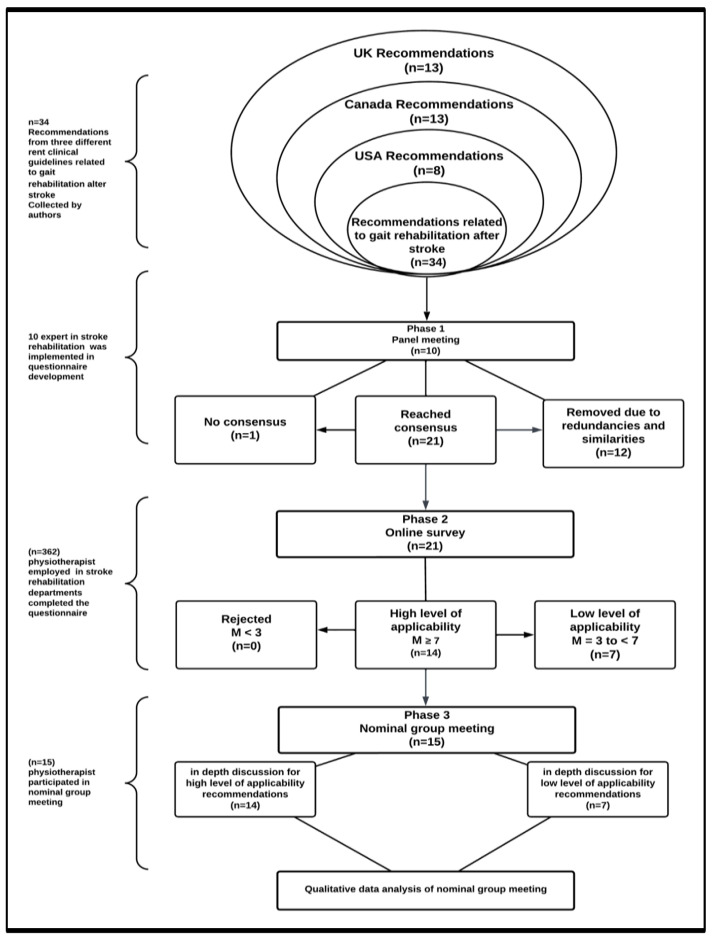
The plan chart outlines the study’s phases. The consensus approach included three phases: Phase 1, panel meeting (n = 10), online survey; and Phase 3, nominal group meeting (n = 15).

A mixed-method study was used. A consensus approach was employed to explore tailoring within the Knowledge-to-Action (KTA) framework. The methodology consisted of three phases: a panel, a survey, and a nominal group meeting (NGM) (Figure 2).

The decision to use the KTA framework over other implementation theories stems from its inclusion of the key concept of tailoring in the knowledge creation process. The consensus approach was selected to deepen the understanding of how tailoring functions and its relevance in stroke rehabilitation, while also exploring the various factors that affect the implementation process.

The continuation of the study received approval from the local research ethics commission (LREC) at the University of Tabuk. All participants provided informed consent before engaging in the study.

### 2.1. Phase 1: The Panel Phase

The authors independently collected the post-stroke gait disorder recommendations from the US, UK, and Canadian stroke guidelines. They collected 34 recommendations from the US, UK, and Canadian stroke guidelines for panel voting to determine their applicability to stroke-related gait disorder rehabilitation.

#### 2.1.1. The Sample Size

The panel group consisted of 7 clinicians and 3 academics; all recruited from local hospital systems and universities. The inclusion criteria required that participants be either physiotherapists with expertise in stroke rehabilitation working in hospitals or academics who possess extensive experience in stroke rehabilitation and integrate it into their university curriculum.

#### 2.1.2. The Procedure

A projector displayed all recommendations during the panel meeting. Participants were instructed to organize the recommendations as needed before voting on their relevance and suitability for stroke rehabilitation using a two-point Likert scale: “Yes, it helps with gait disorders rehabilitation after stroke” and “No, it doesn’t help with gait disorders rehabilitation after stroke”.

#### 2.1.3. Data Analysis

**Figure 3:**
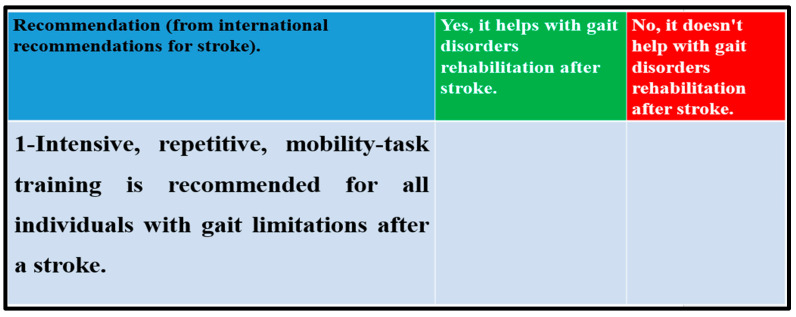
An illustration of a panel meeting where members are voting on the feasibility of implementing a specific recommendation for gait rehabilitation after a stroke.

Any recommendation that received at least 70% approval from the votes was included, while those that did not meet this threshold were excluded based on the consensus findings [18] (Figure 3). The survey for the next phase was developed based on the consensus findings.

### 2.2. Phase 2: The Survey

The goal of this survey was to find out what specialized stroke physiotherapists think about how realistic it is to apply the agreed-upon recommendations from Phase 1 (the panel meeting) in actual stroke rehabilitation settings.

#### 2.2.1. The Sample Size

The online survey was distributed to 598 participants. The survey exclusively comprised physiotherapists in Saudi Arabia who met the eligibility criteria of having a minimum of one year of experience in stroke rehabilitation. The study excluded physiotherapists who were not actively involved in stroke rehabilitation practice.

#### 2.2.2. The Procedure

**Figure 4:**
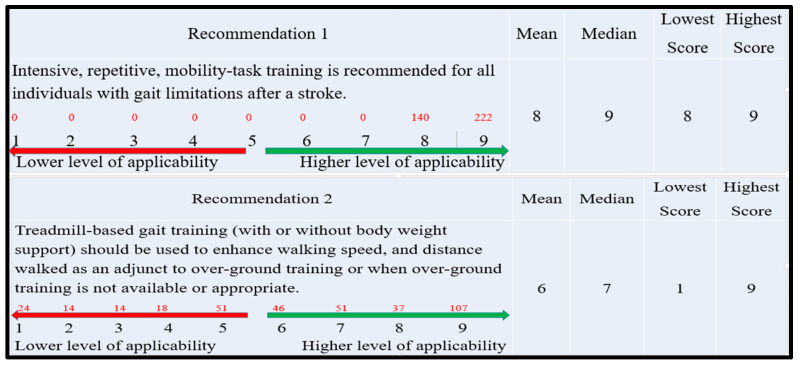
This figure illustrates an example of recommendations included in a survey distributed to physiotherapists. Participants were asked to rate these recommendations on a scale from 1 to 9 to indicate their level of agreement with each item listed in the survey. Recommendation 1 achieved consensus with narrow variations between the lowest and highest scores, while recommendation 2 achieved consensus with significant variations between the lowest and highest scores. (Red numbers indicate the frequency of responses among participants.).

Each recommendation was rated on a Likert scale from 1 to 9, with 1 indicating the lowest level of agreement and 9 indicating the highest level of agreement [19] (Figure 4). We distributed questionnaires generated by Google Forms to Saudi physiotherapists via social networks to increase the number of eligible responses. Participation in the questionnaire required an email address. Consequently, all participants were sent an invitation, a cover letter, and a form of consent.

#### 2.2.3. Data Analysis

The Statistical Package for the Social Science version 25 for windows (SPSS Version25; IBM, Armonk, NY, USA) was employed to calculate the frequency, median, and interquartile range for each recommendation. The absence of consensus made recommendations with a median score below 3 unacceptable. Recommendations with a median score of three or above were chosen for the nominal group discussion (phase 3) [19].

### 2.3. Phase 3: Nominal Group Meeting

This phase involved analyzing the survey results from phase 2 to discuss participants’ ratings of the recommendations. The objective was to identify and deliberate on recommendations that achieved consensus, defined as a median score of 7 or above, as well as those that did not reach consensus, indicated by a median score below 3. Additionally, it is important to explore why some recommendations attained consensus despite exhibiting significant score variations (for instance, with a lowest score of 1 and a highest score of 9), while others showed only minor variations (for example, with a lowest score of 8 and a highest score of 9).

#### 2.3.1. The Sample Size

During this phase, 15 stroke rehabilitation physiotherapists who had completed the survey (phase 2) expressed interest in a nominal group meeting.

#### 2.3.2. The Procedure

Before starting the discussion, the questionnaire findings were shown on screen. The NGM guide featured open-ended and probing questions specifically designed to align with the study’s objectives. These questions aimed to gather essential information required to achieve the study’s goals (Appendix A). Fifteen stroke rehabilitation physiotherapists who completed the questionnaire expressed interest in an NGM to discuss Phase 2’s findings.

Braun and Clarke’s [20] thematic analysis is performed manually and comprises six distinct phases: (1) familiarization, which entails actively reading the data to understand its depth and breadth; (2) coding, in which the researcher identifies compelling and significant pieces of data that may contribute to theme development; (3) linking the identified themes to all coded data; (4) examining the coded data to develop cohesive and refined themes; (5) identifying topic titles along with the associated data; and (6) reporting, which involves writing the final analysis and detailing the identified themes (Appendix B).

#### 2.3.3. Data Analysis

The transcripts were analyzed for themes. Two researchers, focusing on data coding, carefully reviewed the transcriptions from the NGM. They entered the transcriptions in Excel files for the coding process. Following this, the researchers independently identified the main themes and subthemes.

To create initial themes and subthemes, begin by identifying patterns of meaning within the codes. Establish subthemes. Next, analyze the subthemes to identify overarching concepts. Finally, verify the proposed themes against the dataset to assess how accurately they represent the data. Participant numbers (P#s) were assigned to maintain anonymity.

## 3. Results

Understanding physiotherapists’ viewpoints, assessing their ratings of the recommendations to reach consensus, and analyzing the discussions from the notional group meeting may aid in identifying potential barriers and supports to consider during the implementation of the final recommendation regarding gait disorders after stroke.

### 3.1. Result of Phase 1: Panel Meeting

We provided a comprehensive set of 34 recommendations for panel voting to determine their applicability to stroke-related gait disorder rehabilitation. Twelve recommendations were eliminated due to their duplication with the stroke recommendations from the US, UK, and Canada. Table 1 displays the characteristics of the panel meeting participants.

After eliminating duplicate recommendations, a total of 21 recommendations achieved a consensus level (70%), and one recommendation fell short of achieving a consensus level based on the panel’s voting.

### 3.2. Result of Phase 2: Online Survey

In this phase, out of the 598 participants in the survey, 362 provided complete data (Table 2). In total, 14 of 21 stroke rehabilitation gait recommendations were highly useful (with a median score of 7 or higher). Only seven recommendations obtained a median score of 3–7, indicating minimal applicability. No recommendation was rejected (Appendix C).

### 3.3. Results of Phase 3: Nominal Group Meeting (NMG)

Fifteen stroke rehabilitation physiotherapists (n = 15) who completed the survey in phase 2 expressed interest in participating in a nominal group meeting. The characteristics of the participants in this phase are presented in Table 3.

In this phase, we analyzed the online survey findings that had previously been disrupted in phase 2. Four main themes and nine subthemes were derived from the NMG (Table 4). This analysis aims to clarify why some recommendations reached a consensus level while others did not. This phase’s findings may shed light on why participants modified the recommendations before implementing them in their practice.

#### 3.3.1. Organizational Factors

All participants agreed that organizational issues could impact the tailoring of rehabilitation evidence for stroke gait disorders. In subtheme 1 (clinical context), participants said that the resources available at work—like money, staff, time, and equipment—were very important in deciding whether to use rehabilitation recommendations for stroke gait disorders in their everyday jobs. They noted that the resources of healthcare facilities could influence the implementation of these recommendations.

“I recognize the significance of this recommendation for stroke gait rehabilitation; however, my department does not have the necessary equipment to implement it. I voted 5 out of 9.”

We noted a considerable consensus among recommendations that did not require equipment, in contrast to those that did. For example, several recommendations received a median score of 9.

“This recommendation does not require any equipment; it depends instead on the professional’s skill. In my opinion, these comments may help clarify the rationale for achieving a median value of 9.”

Participants concurred that organizational culture (subtheme 2) significantly influences the tailoring of clinical practice recommendations. Key elements of this culture include strong leadership, staff training, effective communication among healthcare professionals, teamwork, and feedback from clinical experiences.

“I rated some recommendations based on departmental teamwork and communication. This fosters knowledge exchange, which contributes to the development of optimal practices.”

Participants in subtheme 3 (Organizational policies) emphasized a conflict between the desire to tailor recommendations and the need to adhere to organizational policies. While participants believe that personalized recommendations could benefit their patients, this conflict may impede the practical implementation of those suggestions, as they may not conform to established organizational guidelines.

“In my opinion, certain recommendations may be feasible and could be implemented”. However, rigid organizational policies can obstruct the effective application of evidence in real-world clinical settings.”

#### 3.3.2. Individual Clinician Factors

Participants emphasized the importance of considering individual factors when tailoring recommendations for rehabilitating gait disorders in stroke patients. They noted that their knowledge, skills, experience, and training significantly influenced how they ranked the recommendations in the survey (Phase 2). This reliance on personal expertise may explain the variability in consensus ratings among participants regarding specific recommendations. One participant remarked,

“Individuals lacking sufficient expertise, practical experience, skills, and training may have a limited understanding of recommendation effectiveness in producing favorable results with patients.”

Those in the “clinical experience and expertise” group said that their past use of the recommendations and their understanding of how to use them in real-life situations influenced their choice to apply them in helping patients with stroke-related walking problems. As one participant stated,

“If the recommendation is not being utilized, it is unlikely that one would use it on a daily basis.”

#### 3.3.3. Patient Factors

The third theme explored patient variables that could potentially impact recommendations for the rehabilitation of gait disorders following a stroke. The first subtheme focused on how various health conditions can affect clinical practice recommendations. Participants noted that different medical concerns and comorbidities can influence the tailoring of recommendations. One participant stated,

“Before considering the implementation of any recommendations for my patient, I took into account medical problems related to strokes, as well as memory and cognitive issues that affect individuals who have experienced a stroke.”

Participants in the second subtheme highlighted the importance of patient engagement and shared decision-making in physiotherapy practice. They emphasized that when physiotherapists engage in shared decision-making with patients, it can lead to improved health outcomes, increased patient satisfaction, and more personalized treatment approaches. One participant remarked,

“When deciding which recommendations to incorporate into my practice, I considered how I could effectively engage with my patients in meaningful discussions and provide clear explanations about this recommendation to ensure that interventions align with their values, preferences, and goals.”

#### 3.3.4. Other Factors

The findings suggest that additional factors may influence participants’ decisions regarding the implementation of certain recommendations in their clinical practice. The first subtheme is titled “Research of RCT Design”. Participants noted that research methodologies, such as randomized controlled trials (RCTs), may not be applicable to all patients and can be difficult to implement. The stringent protocols of RCTs can limit the ability to tailor interventions to align with each patient’s specific needs, preferences, and treatment responses, ultimately diminishing their effectiveness. Several participants talked about the importance of employing qualitative research methods to collect empirical data from healthcare professionals, patients, and caregivers involved in rehabilitation for gait disorders following a stroke. One participant noted,

“The strict inclusion and exclusion criteria of a randomized controlled trial (RCT) may exclude the patients I am trying to treat. Therefore, increasing the use of qualitative methods may be beneficial.”

Client-related factors associated with the stages of stroke also affected how participants tailored their recommendations (the second subtheme). In the initial phase of a stroke, the primary focus is on preventing further complications. In contrast, patients in the later stages may require more intensive and specialized rehabilitation aimed at addressing functional impairments. One participant explained how this insight could influence the tailoring of specific clinical practice recommendations, stating,

“For me, in the early stages, I occasionally focused on the prevention of secondary complications, whereas in the late stages, I used to focus on the improvement of motor function.”

## 4. Discussion

This study examined the factors that may affect the tailoring of gait disorder recommendations based on international stroke guidelines. Several factors require investigation to evaluate their impact on the effectiveness of this tailoring process:

### 4.1. Organizational Factors

The findings indicate that the implementation of recommendations within a specific organization relies on several key factors: funding, staffing, time management, leadership, ongoing training and education, communication, and policies. These elements significantly influence the range of professional activities permitted within the organization. The results support previous research, which demonstrates the value of considering specific requirements, priorities, regulations, and resources when customizing recommendations for clinical practice [21,22]. Prior studies suggest that assessing user perspectives, available resources, daily routines, local leadership, opinion leaders, and systems is essential for effectively adapting knowledge to local contexts [21,22]. This research highlights that successful implementation of changes is more likely in organizations that prioritize evidence-based planning, promote open discussions, and allocate resources effectively.

### 4.2. Individual Clinician Factors

The findings of this study indicate that specific clinician characteristics significantly influence the tailoring and implementation of evidence in clinical settings. The effectiveness of tailored recommendations in clinical practice is heavily dependent on clinical skills, experience, knowledge, beliefs, and attitudes [14,23,24]. These factors are essential for integrating recommendations into patient-centered healthcare

Consistent with this study’s findings, several researchers argue that involving end users in the tailoring of evidence-based interventions to fit their specific context has proven to be effective [14,22,23]. Additionally, when end users are not engaged in the tailoring process, it often results in inadequate tailoring rates and negatively affects outcome quality [23].

This research emphasizes the value of end-user feedback in the tailoring process [14,23]. By exploring professional attitudes, organizations can gain a deeper understanding of how to implement and sustain evidence-based practices [14,23,24]. Pereira et al. demonstrated that modifying implementation strategies to address locally recognized barriers and facilitators can enhance the acceptance of recommendations and improve care interventions [22].

### 4.3. Patient Factors

This study found that the physical, cognitive, and psychological abilities of stroke patients significantly influenced rehabilitation recommendations for gait disorders. The evidence suggests that individuals with multiple comorbidities or complex medical histories may require a personalized approach, as national recommendations may not fully address their specific clinical situations [23,24]. Several authors emphasized the importance of thoroughly evaluating potential treatment options and their feasibility for individual patients before applying recommendations in clinical practice [25]. Additionally, the study’s findings highlighted that patient engagement is crucial in the physiotherapists’ decision-making process regarding the application of national recommendations. Aligning evidence-based practices with the patient’s goals, values, and preferences is essential for effective implementation. Shared decision-making is vital to ensure that the treatment plan aligns with the patient’s priorities [25,26].

### 4.4. Other Factors

Feasibility refers to the practicality of proposed evidence for implementation, specifically how well it aligns with current practices [27]. The study’s results showed that the practicality of the recommendations from a randomized controlled trial (RCT) significantly impacted how they were used to treat walking problems after a stroke [26,27,28]. This challenge underscores the concept of feasibility. The research suggested that physicians may need to incorporate information from various sources, such as observational studies, patient registries, and real-world evidence, alongside RCTs to better tailor treatment plans for stroke management [27,28]. Furthermore, employing adaptive trial designs, personalized outcome measures, and considering patient preferences and goals can help bridge the gap between RCT evidence and the unique needs of stroke patients [25,26,27].

Therefore, recommendations must be tailored to fit within existing delivery models in the targeted setting [26]. The importance of feasibility, in terms of practical and organizational factors, has been emphasized as crucial for effectively translating research findings into sustainable healthcare delivery and improved patient outcomes. By focusing on feasibility, we can ensure the optimal implementation of evidence. However, some authors have expressed concerns about the difficulty of maintaining accuracy when adapting evidence-based principles to specific settings [26,28]. Excessive modification of these recommendations can result in a loss of their original meaning, potentially negatively impacting the quality of care and its outcomes.

## 5. Study Limitations

All participants in this study were Saudi physiotherapists, indicating that their expertise is specific to the context of Saudi healthcare. As a result, the research findings may only be applicable to similar environments. The primary objective of the study was to tailor stroke guidelines to address specific issues, such as gait impairments, rather than to generalize findings statistically. Furthermore, rehabilitation center managers played a role in recruiting stroke physiotherapists during the data collection phase, which may have created a sense of obligation for participants to take part in the study. Communication with stroke physiotherapists also required prior approval from the management of the rehabilitation centers. Another limitation of this study is its inability to evaluate how well individuals and organizations adhere to the established recommendations. There is a need for international collaboration.

## 6. Conclusions

The KTA framework incorporates tailoring as a key element of its funnel structure. The key to successful tailoring is that there is a match between the expected research findings, the targeted knowledge users, and the knowledge translation strategies selected. Furthermore, essential issues should be explored before tailoring recommendations from international clinical guidelines to a specific issue. This includes examining organizational, individual, and patient factors, engaging end users, and assessing feasibility. This study demonstrates how a systematic approach, such as the consensus method, can effectively facilitate the conversion of research knowledge into practical applications. However, it is necessary to account for various factors to successfully tailor international recommendations that align with real-world practices.

## Figures and Tables

**Table 1:** Professional characteristics of the participants in the panel’s meeting (Phase 1).

Current Job Title	Number of Participants	Background
Clinical senior physical therapist	4	Stroke rehabilitation
Clinical consultant of rehabilitation	3	Stroke rehabilitation
Professor of stroke rehabilitation	1	Stroke rehabilitation
Professor of stroke and older people’s care	1	Stroke rehabilitation and falls after stroke.
Professor of neurorehabilitation.	1	Exercise interventions after neurological diseases.

**Table 2:** The sociodemographic data of physiotherapist respondents.

Q1: Gender	Frequency (n)	Valid Percentage
Male	189	52.2%
Female	173	47.8%
Total	362	100%
Q2: Age		
20–29	228	63%
30–39	108	29.8%
40–49	21	5.8%
≥50	5	1.4%
Total	362	100%
Q3: Qualification		
Diploma	4	1.1%
BSc	249	69.8%
MSc	101	27.9%
PhD	8	2.2%
Total	362	100%
Q4: experience		
1–5 years	250	69.1%
6–10 years	40	11%
11–15 years	32	8.8%
≥15 Years	40	11%
Total	362	100%
Q5: Patients/day		
1–5 patients	302	83.4%
6–10 patients	23	6.4%
11–15 patients	14	3.9%
≥15 patients	23	6.4%
Total	362	100%
Q6: Region		
Central	146	40.3%
East	63	17.4%
West	82	22.7%
North	30	8.3%
South	41	11.3%
Total	362	100%
Q7: Workplace		
Governmental (hospital\center)	188	51.9%
Private (hospital/center)	174	48.1%
Total	362	100%

**Table 3:** Characteristics of participants in the nominal group meeting (n = 15).

Categories		Numbers (n, %)
Gender	Male	11 (73.3%)
	Female	4 (26.7%)
Age	20–25	1 (4%)
	26–30	4 (26.7%)
	31–35	3 (20%)
	36–40	5 (33.3%)
	≥41	2 (13.3%)
Qualification	Diploma	0 (0%)
	Bsc.	8 (53.3%)
	DPT	2 (13.3%)
	Master	3 (20%)
	PhD	2 (13.3%)
Number of years working with people with stroke	1–5 Years	4 (26.7%)
	6–10 Years	6 (40%)
	11–15 Years	3 (20%)
	≤16 Years	2 (13.3%)

**Table 4:** Main themes and subthemes.

Theme	Subtheme
Theme 1: Organizational factors	Subtheme 1: Clinical context. Subtheme 2: Organizational culture. Subtheme 3: Organizational policies.
Theme 2: Individual clinician factors	Subtheme 1: Clinical experience and expertise. Subtheme 2: Skill, knowledge, and attitudes.
Theme 3: Patient factors.	Subtheme 1: Multimorbidity and comorbidities. Subtheme 2: Patient engagement and shared decision-making.
Theme 4: Other factors.	Subtheme 1: Research on RCT design. Subtheme 2: Stroke phases.

## Data Availability

All data generated or analyzed during this study are included in this published article.

## Appendix A. Nominal Group Meeting Guide

Introduction, process explanation, and preliminary information.

- Thank participants for participating and explain the purpose of the Study.
- Introduce self.
- Explain the nominal group meeting process.

During this meeting, we will present you with the results of the survey. However, our objective is to review a selection of the recommendations on which you all reached a consensus, some of the recommendations where the recommendations were applicable, recommendations where you all agreed that the recommendations were not relevant, and two or three recommendations where you just could not reach a consensus. The purpose of this meeting is to gain insight into the reasoning behind your assignment of a consensus score to each recommendation.

I am going to lead the interview (SF). We would like to record this to give us some data to, as I said, sort of understand how people go about adapting national clinical guidelines. So my colleague (SA) will have circulated an information sheet and a consent form. All of the usual caveats apply, so nobody will know that you have participated in this interview. We will report quotations from the interview meeting, but we will not attribute them to any specific individual within this group. We will store the data in a secure location and destroy it after the study is complete. Please feel free to answer or ask any questions about what we need to do today.

Part 1 questions

A couple of the recommendations for incorporation lacked consensus. Consensus refers to the requirement that the recommendation’s median score be seven or greater. The first group of chosen recommendations did not reach a consensus on their inclusion in the guidelines for managing gait disorders after a stroke. Considering the significant differences among all of you as a group, I believe it would be advantageous to begin at this point and discuss each specific recommendation for every individual. We have several questions about these particular recommendations that we’d like to ask you. Kindly provide an indication of the extent to which biofeedback aligns with your present practices. How closely or distantly does biofeedback correspond to your current activities? Does anyone exhibit this behavior in this context, or does it go completely unnoticed?

Part 2 questions

In this section, we calculated the median score for these recommendations, which was interesting. In this section of our discussion, we will concentrate on recommendations that garnered a robust consensus for their inclusion in the management of gait disorders following stroke. We wondered why you think there might have been such high consensus, and why perhaps a handful of people fully agreed to include these kinds of recommendations in their practice in gait disorders rehabilitation after stroke? What caused the participants to show little variance in their practice of including or excluding these types of suggestions for gait abnormalities after stroke?

Part 3 questions

In this section, our focus will be on recommendations that have received ratings falling within a range of consensus to rejection. This indicates that the recommendations haven’t reached a sufficient level of consensus or rejection.

## Appendix B. Final Codes

**#**	**Code**	**Description**
1	Patient characteristics	Individual patient characteristics such as age, sex, medical history, comorbidities, and genetic factors can influence the tailoring of evidence-based practices. Different patient populations may respond differently to treatments, so considering these factors is essential in tailoring evidence to meet individual patient needs.
2	Clinical context	The specific clinical context, including the healthcare setting, available resources, and local guidelines, can influence the tailoring of evidence. The feasibility and applicability of certain interventions may vary depending on the resources and infrastructure available in a particular healthcare setting.
3	Disease severity and stage	The severity and stage of the disease can impact the tailoring of evidence-based practices. The evidence supporting a particular intervention may differ based on the disease’s severity and stage, and treatment decisions may need to be adjusted accordingly.
4	Coexisting conditions	Patients often have multiple medical conditions, and the presence of coexisting conditions can influence treatment decisions. Tailoring evidence-based practices requires considering the impact of treatment on all coexisting conditions to ensure the best outcomes for the patient.
5	Patient preferences and values	Patient preferences, values, and goals play a crucial role in tailoring evidence-based practices. Shared decision-making between healthcare providers and patients is important to understand patient preferences and incorporate them into the treatment plan.
6	Emerging research	Clinical practice is dynamic, and new research findings continuously emerge. As new evidence becomes available, it may prompt modifications in existing practices. Staying updated with the latest research and integrating it into clinical decision-making is essential for tailoring evidence-based practices.
7	Clinical expertise	The clinical expertise and experience of healthcare providers are vital in tailoring evidence-based practices. Providers need to integrate their knowledge and experience with the available evidence to develop individualized treatment plans for patients.
8	Cost and resource considerations	The cost-effectiveness and availability of resources can influence the tailoring of evidence-based practices. In resource-limited settings, healthcare providers may need to adapt interventions to make them more feasible and cost-effective.
9	Cultural and societal factors	Cultural and societal factors can influence the tailoring of evidence-based practices. Medical practices and beliefs may vary across different cultures and communities, and healthcare providers need to consider these factors when tailoring evidence to individual patients. Cultural beliefs, values, and preferences can impact treatment decisions and adherence to therapies.
10	Risk-benefit assessment	The assessment of risks and benefits associated with a particular intervention is crucial in tailoring evidence-based practices. Healthcare providers need to evaluate the potential benefits of a treatment against the risks and potential harms, taking into account individual patient characteristics and circumstances.
11	Guidelines and recommendations	Clinical practice guidelines and recommendations provided by professional medical organizations are important sources of evidence-based information. These guidelines often synthesize the available evidence and provide recommendations for specific conditions or interventions. Healthcare providers may use these guidelines as a framework for tailoring evidence-based practices.
12	Patient education and health Literacy	Tailoring evidence-based practices requires effective communication and patient education. Health literacy, the ability of patients to understand and utilize health information, plays a critical role in the implementation of evidence-based practices. Healthcare providers should ensure that patients have access to accurate and understandable information to make informed decisions about their care.
13	Health system factors	The structure and organization of the healthcare system can influence the tailoring of evidence-based practices. Factors such as reimbursement policies, healthcare regulations, and the availability of electronic health records can impact the implementation and customization of evidence-based practices within a healthcare system.
14	Time and resource constraints	Healthcare providers often face time constraints in clinical practice. The availability of resources, including time for patient consultations and access to necessary diagnostic tests and treatments, can impact the tailoring of evidence-based practices. Providers need to balance the available resources with the best available evidence to provide optimal care within the given constraints.
15	Clinical trial limitations	The evidence base for clinical practice is primarily derived from clinical trials and research studies. However, clinical trials have limitations, such as specific inclusion and exclusion criteria, limited sample sizes, and relatively short follow-up periods. Healthcare providers need to consider these limitations when tailoring evidence-based practices and should be cautious about generalizing the findings to diverse patient populations.
16	Ethical considerations	Ethical principles, such as autonomy, beneficence, non-maleficence, and justice, play a significant role in tailoring evidence-based practices. Healthcare providers need to consider the ethical implications of treatment decisions, respecting patient autonomy and ensuring that the chosen interventions align with ethical standards.
17	Patient outcomes and preferences	The desired outcomes and preferences of individual patients are crucial in tailoring evidence-based practices. Healthcare providers need to consider what outcomes are most important to the patient, such as symptom relief, functional improvement, quality of life, or long-term survival. Understanding patient preferences allows for shared decision-making and the customization of treatments accordingly.
18	Feedback and continuous learning	Tailoring evidence-based practices requires a commitment to continuous learning and improvement. Healthcare providers should actively seek feedback from patients, colleagues, and interdisciplinary teams to evaluate the effectiveness and impact of interventions. This feedback loop helps refine and adapt practices over time to optimize patient outcomes.
19	Patient safety and risk management	Patient safety is a fundamental consideration when tailoring evidence-based practices. Healthcare providers need to assess and mitigate potential risks associated with interventions, including adverse effects, drug interactions, and medical errors. Incorporating risk management strategies into the tailoring process ensures patient safety and minimizes harm.
20	Interdisciplinary collaboration	Tailoring evidence-based practices often requires collaboration among healthcare professionals from different disciplines. Interdisciplinary teamwork allows for a comprehensive assessment of patient needs and the integration of diverse perspectives and expertise. Collaborative decision-making can lead to more effective tailoring of evidence-based practices.
21	Patient advocacy and empowerment	Patient advocacy and empowerment are crucial in the tailoring of evidence-based practices. Encouraging patients to actively participate in their care, providing education and resources, and supporting their autonomy and decision-making process empower patients to make informed choices and actively engage in their treatment.
22	Health policy and reimbursement systems	Health policy and reimbursement systems can shape the tailoring of evidence-based practices. National or local healthcare policies, insurance coverage policies, and reimbursement mechanisms may impact the availability and accessibility of certain interventions. Healthcare providers need to navigate these systems to ensure appropriate reimbursement and optimize patient access to evidence-based care.
23	Education and training	Education and training of healthcare providers are critical in the tailoring of evidence-based practices. Continuous professional development ensures that healthcare providers stay updated with the latest evidence, guidelines, and best practices. Training programs can also focus on enhancing skills in critical appraisal of evidence, shared decision-making, and the application of tailored approaches in clinical practice.
24	Patient engagement and shared decision-making	Patient engagement and shared decision-making are integral to the tailoring of evidence-based practices. Involving patients in the decision-making process promotes patient-centered care and ensures that interventions align with their values, preferences, and goals. Shared decision-making facilitates the customization of evidence-based practices to meet the unique needs of each patient.
25	Feedback from clinical experience	Clinical experience and feedback play a vital role in refining and tailoring evidence-based practices. Healthcare providers continuously learn from their own experiences and the experiences of colleagues. They can adapt and modify interventions based on real-world outcomes, patient responses, and emerging evidence to optimize the tailoring of evidence-based practices.
26	Multimorbidity and comorbidities	Many patients have multiple chronic conditions, known as multimorbidity, or comorbidities, where they have one or more additional health conditions alongside their primary condition. Tailoring evidence-based practices becomes complex in such cases as the management of one condition may affect the management of others. Healthcare providers need to carefully consider the interactions and potential conflicts among treatments to ensure optimal outcomes and minimize harm.
27	Health behavior and psychological factors	Tailoring evidence-based practices requires considering the health behaviors and psychological factors that influence patient engagement and adherence to treatments. Factors such as motivation, beliefs, social support, and mental health can impact a patient’s ability to follow recommended interventions. Healthcare providers should assess and address these factors to enhance treatment outcomes.
28	Interprofessional collaboration	Collaborative teamwork among healthcare professionals is crucial in tailoring evidence-based practices. Interprofessional collaboration involves communication, coordination, and shared decision-making among professionals from different disciplines, such as physicians, nurses, pharmacists, and allied health professionals. This collaboration allows for a comprehensive assessment of patient needs and the integration of diverse perspectives in tailoring care.
29	Environmental and occupational factors	Tailoring evidence-based practices may involve considering environmental and occupational factors that impact a patient’s health. For example, healthcare providers may need to consider workplace conditions, or access to clean air and water when tailoring interventions. Addressing these factors can contribute to improved health outcomes and the prevention of occupational and environmental diseases.
30	Health system resources and constraints	Tailoring evidence-based practices can be influenced by the available resources and constraints within the healthcare system. Factors such as limited funding, workforce shortages, time constraints, and competing priorities can impact the extent to which practices can be tailored. Healthcare providers must navigate these challenges and find ways to optimize tailoring within the resource constraints.
	Clarifying question	Clarification on a certain issue in question.
	Unrelated discussion	The discussion presented is not directly relevant to the research questions at hand.

## Appendix C. The Degree of Agreement (Consensus) on Recommendations

**Recommendation 1**									**Mean**	**Median**	**Lowest** **Score**	**Highest** **Score**
Intensive, repetitive, mobility-task training is recommended for all individuals with gait limitations after a stroke.									8	9	8	9
0	0	0	0	0	0	0	140	222				
1	2	3	4	5	6	7	8	9				
Lower level of applicability					higher level of applicability							

**Recommendation 2**									**Mean**	**Median**	**Lowest** **Score**	**Highest** **Score**
Treadmill-based gait training (with or without body weight support) should be used to enhance walking speed, and distance walked as an adjunct to over-ground training or when over-ground training is not available or appropriate.									6	7	1	9
24	14	14	18	51	46	51	37	107				
1	2	3	4	5	6	7	8	9				
lower level of applicability					higher level of applicability							

**Recommendation 3**									**Mean**	**Median**	**Lowest** **Score**	**Highest** **Score**
Mechanically assisted walking (treadmill, electromechanical gait trainer, robotic device, servo-motor) with body weight support may be considered for patients who are nonambulatory or have low ambulatory ability early after stroke.									5	5	1	9
46	23	37	28	56	24	46	46	56				
1	2	3	4	5	6	7	8	9				
lower level of applicability					higher level of applicability							

**Recommendation 4**									**Mean**	**Median**	**Lowest** **Score**	**Highest** **Score**
Robot-assisted movement training to improve motor function and mobility after stroke in combination with conventional therapy may be considered									5	5	1	9
56	33	14	37	42	14	37	50	79				
1	2	3	4	5	6	7	8	9				
lower level of applicability					higher level of applicability							

**Recommendation 5**									**Mean**	**Median**	**Lowest** **Score**	**Highest** **Score**
People with stroke who are able to walk (albeit with the assistance of other people or assistive devices) and who wish to improve their mobility at any stage after stroke should be offered access to equipment to enable intensive walking training such as treadmills or electromechanical gait trainers. To achieve this, training needs to be at 60-85% heart rate reserve (by adjustment of inclination or speed) for at least 40 min, three times a week for 10 weeks.									4	5	1	9
81	23	14	23	79	22	13	41	66				
1	2	3	4	5	6	7	8	9				
lower level of applicability					higher level of applicability							

**Recommendation 6**									**Mean**	**Median**	**Lowest** **Score**	**Highest** **Score**
Practice walking with either a treadmill (with or without body-weight support) or overground walking exercise training combined with conventional rehabilitation may be reasonable for recovery of walking function.									7	8	1	9
9	9	28	14	18	18	74	37	155				
1	2	3	4	5	6	7	8	9				
lower level of applicability					higher level of applicability							

**Recommendation 7**									**Mean**	**Median**	**Lowest** **Score**	**Highest** **Score**
incorporating cardiovascular exercise and strengthening interventions is reasonable to consider for recovery of gait capacity and gait-related mobility tasks.									7	8	1	9
9	5	14	28	23	23	70	70	120				
1	2	3	4	5	6	7	8	9				
lower level of applicability					higher level of applicability							

**Recommendation 8**									**Mean**	**Median**	**Lowest** **Score**	**Highest** **Score**
Biofeedback, in the form of visual and/or auditory signals to indicate unequal weight bearing and timing, can be used to enhance gait training and improve functional recovery.									8	7	8	9
0	0	0	0	0	0	0	232	130				
1	2	3	4	5	6	7	8	9				
lower level of applicability					higher level of applicability							

**Recommendation 9**									**Mean**	**Median**	**Lowest** **Score**	**Highest** **Score**
Virtual reality training (such as non-immersive technologies) could be considered as an adjunct to conventional gait training.									5	5	1	9
13	5	28	42	28	28	51	51	115				
1	2	3	4	5	6	7	8	9				
lower level of applicability					higher level of applicability							

**Recommendation 10**									**Mean**	**Median**	**Lowest** **Score**	**Highest** **Score**
Rhythmic auditory stimulation (RAS) should be considered for improving gait parameters in stroke patients, including gait velocity, cadence, stride length, and gait symmetry.									5	6	1	9
41	14	20	46	37	51	51	32	70				
1	2	3	4	5	6	7	8	9				
lower level of applicability					higher level of applicability							

**Recommendation 11**									**Mean**	**Median**	**Lowest** **Score**	**Highest** **Score**
Group therapy with circuit training is a reasonable approach to improve walking.									8	9	8	9
0	0	0	0	0	0	0	138	224				
1	2	3	4	5	6	7	8	9				
lower level of applicability					higher level of applicability							

**Recommendation 12**									**Mean**	**Median**	**Lowest** **Score**	**Highest** **Score**
Mental Practice should be considered as an adjunct to lower extremity motor retraining									6	7	1	9
18	9	32	23	47	47	47	56	83				
1	2	3	4	5	6	7	8	9				
lower level of applicability					higher level of applicability							

**Recommendation 13**									**Mean**	**Median**	**Lowest** **Score**	**Highest** **Score**
People with stroke with limited ankle/foot stability or limited dorsiflexion (‘foot drop’) that impedes mobility or confidence should be offered an ankle-foot orthosis (using a lightweight, flexible orthosis in the first instance) or functional electrical stimulation to improve walking and balance, including referral to orthotics if required.									7	8	1	9
14	0	23	14	46	23	46	41	155				
1	2	3	4	5	6	7	8	9				
lower level of applicability					higher level of applicability							

**Recommendation 14**									**Mean**	**Median**	**Lowest** **Score**	**Highest** **Score**
Any orthosis or electrical stimulation device should be evaluated and individually fitted before long-term use.									8	8	8	9
0	0	0	0	0	0	0	184	178				
1	2	3	4	5	6	7	8	9				
lower level of applicability					higher level of applicability							

**Recommendation 15**									**Mean**	**Median**	**Lowest** **Score**	**Highest** **Score**
People using an orthosis after stroke should be educated about the risk of pressure damage from their orthosis, especially if sensory loss is present in addition to weakness. Services should provide timely access for orthotic repairs and adaptations.									8	8	8	9
0	0	0	0	0	0	0	189	173				
1	2	3	4	5	6	7	8	9				
lower level of applicability					higher level of applicability							

**Recommendation 16**									**Mean**	**Median**	**Lowest** **Score**	**Highest** **Score**
Functional electrical stimulation (FES) should be used to improve strength and function (gait) in selected patients, but the effects may not be sustained.									5	6	1	9
37	15	24	32	60	70	46	60	18				
1	2	3	4	5	6	7	8	9				
lower level of applicability					higher level of applicability							

**Recommendation 17**									**Mean**	**Median**	**Lowest** **Score**	**Highest** **Score**
The person with stroke, their family/carers, and clinicians in all settings should be trained in the safe application and use of orthoses and electrical stimulation devices.									8	8	7	9
0	0	0	0	0	0	101	107	154				
1	2	3	4	5	6	7	8	9				
lower level of applicability					higher level of applicability							

**Recommendation 18**									**Mean**	**Median**	**Lowest** **Score**	**Highest** **Score**
Stroke services should have local protocols and agreements in place to ensure specialist assessment, evaluation, and follow-up is available for long-term functional electrical stimulation use.									6	7	1	9
32	9	14	14	51	46	37	56	103				
1	2	3	4	5	6	7	8	9				
lower level of applicability					higher level of applicability							

**Recommendation 19**									**Mean**	**Median**	**Lowest** **Score**	**Highest** **Score**
People with limited mobility after stroke should be assessed for, provided with, and trained to use appropriate mobility aids, including a wheelchair, to enable safe independent mobility.									7	8	1	9
5	0	9	24	32	18	24	74	176				
1	2	3	4	5	6	7	8	9				
lower level of applicability					higher level of applicability							

**Recommendation 20**									**Mean**	**Median**	**Lowest** **Score**	**Highest** **Score**
People with stroke who are mobile should be assessed for real-world walking such as road crossing, walking on uneven ground, over distances and inclines. This should include assessment of the impact of dual tasking, neglect, vision, and confidence in busy environments.									6	8	1	9
9	5	28	23	37	32	37	32	159				
1	2	3	4	5	6	7	8	9				
lower level of applicability					higher level of applicability							

**Recommendation 21**									**Mean**	**Median**	**Lowest** **Score**	**Highest** **Score**
Clinicians should not use risk assessment protocols that limit training for fear of cardiovascular or other adverse events, given the good safety record of repetitive gait training however it is delivered.									5	6	1	9
56	9	32	28	46	60	37	37	57				
1	2	3	4	5	6	7	8	9				
lower level of applicability					higher level of applicability							
